# A CRISPR-Cas9 sex-ratio distortion system for genetic control

**DOI:** 10.1038/srep31139

**Published:** 2016-08-03

**Authors:** Roberto Galizi, Andrew Hammond, Kyros Kyrou, Chrysanthi Taxiarchi, Federica Bernardini, Samantha M. O’Loughlin, Philippos-Aris Papathanos, Tony Nolan, Nikolai Windbichler, Andrea Crisanti

**Affiliations:** 1 Department of Life Sciences, Imperial College London, South Kensington Campus, London SW7 2AZ, UK; 2 Section of Genomics and Genetics, Department of Experimental Medicine, University of Perugia, 06132 Perugia, Italy; 3 Department of Life Sciences, Imperial College London, Silwood Park, Ascot SL5 7 PY, UK

## Abstract

Genetic control aims to reduce the ability of insect pest populations to cause harm via the release of modified insects. One strategy is to bias the reproductive sex ratio towards males so that a population decreases in size or is eliminated altogether due to a lack of females. We have shown previously that sex ratio distortion can be generated synthetically in the main human malaria vector *Anopheles gambiae*, by selectively destroying the X-chromosome during spermatogenesis, through the activity of a naturally-occurring endonuclease that targets a repetitive rDNA sequence highly-conserved in a wide range of organisms. Here we describe a CRISPR-Cas9 sex distortion system that targets ribosomal sequences restricted to the member species of the *Anopheles gambiae* complex. Expression of Cas9 during spermatogenesis resulted in RNA-guided shredding of the X-chromosome during male meiosis and produced extreme male bias among progeny in the absence of any significant reduction in fertility. The flexibility of CRISPR-Cas9 combined with the availability of genomic data for a range of insects renders this strategy broadly applicable for the species-specific control of any pest or vector species with an XY sex-determination system by targeting sequences exclusive to the female sex chromosome.

Since females largely determine the reproductive capacity of most pest species, a very attractive genetic control strategy is to distort the male-to-female reproductive sex ratio in favour of males, whereby the progressive reduction of females is anticipated to cause a dramatic contraction of the population and eventually its elimination. A synthetic sex-ratio distortion trait has been generated previously in the malaria mosquito *Anopheles gambiae* by expressing during the process of spermatogenesis the His Cys box endonuclease I-PpoI that cleaves a conserved sequence within the ribosomal DNA (rDNA) repeats found solely on the X-chromosome^1^,^2^,^3^. By restricting endonuclease activity to spermatogenesis, X-bearing sperm were selectively destroyed and eggs were predominantly fertilised by Y-bearing sperm destined to produce males^3^. X-shredding, unlike female-killing, operates meiotically therefore no significant reduction in male fertility is incurred. Our results thus provide the foundation for the genetic control of a host of heterogametic pest species with an XY sex-determination system. However, approaches based on the use of the endonuclease I-PpoI can be exclusively applied to a limited number of mosquito species where rDNA genes are located on the X-chromosome. In the vast majority of insects the rDNA genes are not localised on a single X-specific cluster and therefore a more flexible endonuclease platform is needed to enable the targeting of alternative X-chromosome sequences for each species.

Here we describe the first functional CRISPR-Cas9 sex-distortion system (CRISPR^SD^) in the malaria mosquito *A. gambiae*. To demonstrate the feasibility and effectiveness of this approach we utilized the CRISPR-Cas9 nuclease to target an X-linked rDNA sequence that is different from the previously utilized I-PpoI target site and conserved among the malaria vector species of the *A. gambiae* complex: *A. arabiensis*, *A. gambiae*, *A. bwambae*, *A. melas* and *A. merus*^4^,^5^, yet absent from more distantly related insects. The alignment of 28S rDNA sequences revealed a number of potential target regions meeting these criteria and of these, a single guide RNA (gRNA-T1) was chosen for its lack of predicted off-target sites in *A. gambiae* (Fig. 1b, Supplementary Fig. S1).

We designed a germline transformation construct where the Cas9 endonuclease coding sequence was placed under the transcriptional control of the spermatogenesis-specific β2 tubulin promoter^6^. This allowed us to restrict the endonuclease activity to male meiosis despite the likely ubiquitous expression of gRNA-T1 driven by the Pol III promoter of the mosquito U6 snRNA gene^7^,^8^ (Fig. 1a). The CRISPR^SD^ construct was contained within a *piggyBac* transformation vector to allow transposon-mediated insertion into the genome. A total of 4 transgenic lines each containing a unique genomic insertion of the construct were generated as previously described^9^. Single chromosomal integrations were selected and their respective genomic positions determined (Supplementary Table S2).

We then assessed whether male germline expression of the CRISPR^SD^ unit would prevent the transmission of the X-chromosome to their progeny and thereby generate an excess of males. All the CRISPR^SD^ lines showed a strong sex-ratio distortion, with a male bias among progeny ranging from 86.1% to 94.8% of males. The fertility of hemizygous transgenic males was also tested revealing full fertility from three of four strains, with hatching rates between 83.6% and 93.2%, whilst only one strain showed a significant reduction in hatching rate down to an average of 54.3% (Fig. 2, Supplementary Table S2). Residual somatic expression of the CRISPR^SD^ transgene may explain reduced fertility in strain N, probably due to position effects associated with the insertion site (Supplementary Fig. S3). All CRISPR^SD^ strains showed no difference in the average number of eggs laid per mated female when compared to the wild-type control (Fig. 2, Supplementary Table S2). Male germline expression of CRISPR-Cas9 does not appear to suffer markedly from problems related to undesired endonuclease stability and carry-over into the fertilized embryo that had complicated previous attempts to establish sex distortion based on the I-PpoI endonuclease^2^. This is confirmed by another set of experiments in which we find that maternally deposited Cas9 in combination with zygotic expression of the gRNA from the CRISPR^SD^ locus is able to induce embryo lethality but we find no evidence for an effect of paternal carryover of either the gRNA or Cas9 protein on embryo viability (Supplementary Table S2, Supplementary Fig. S4). We confirmed that the observed strong sex distortion phenotype was stably inherited from males to their transgenic sons. For five consecutive generations transgenic males showed consistent levels of male-biased sex ratios in their offspring (Supplementary Table S2). In those rare females that had inherited an X-chromosome from a CRISPR^SD^ father most of the repeats remained susceptible to cleavage, indicating that the vast majority of the rDNA copies retain the intact target sequence (Supplementary Fig. S5). In some individuals a small fraction of the repeats were not cleaved, consistent with the CRISPR-mediated generation of alleles that prevent further cleavage. A similar phenomenon was observed in previous experiments performed on the I-PpoI strains where both molecular and phenotypic analysis confirmed that enzyme-exposed alleles remained susceptible to further cleavage^3^.

In conclusion three of four of the transgenic lines we tested showed high levels of sex ratio distortion as well as full male fertility demonstrating that CRISPR-Cas9 is well suited for the construction of synthetic sex ratio distortion traits. We have demonstrated proof of principle for a flexible system of CRISPR-based sex distorters whose target population can range from a species to a limited subset of genomes without affecting the other members of a sexually reproducing population. The choice of which strategy to employ will have to consider the trade-off of increased likelihood of nuclease-resistant alleles evolving at sites that are less conserved. If function is restored, gene conversion may accelerate this process when targeting multiple repeat arrays.

Two areas of further research remain: First, there is a need to develop a bioinformatic strategy to identify X-linked target sequences in the genomes of insect pest or vector species as such repeats are ipso facto absent from high-quality genome assemblies. Here again, the flexibility of CRISPR-Cas9 may be advantageous as it allows targeting multiple independent target sequences. Multiplexing of gRNAs would also hedge this approach against the rise of cleavage-resistant alleles^10^,^11^,^12^ (Fig. 3b). Secondly, whilst autosomal sex-distorter strains as described herein show high levels of distortion they would require repeated releases to achieve population suppression^13^ (Fig. 3a), e.g. in combination with an inducible expression system which would need to be established in each target species. However, genetic linkage of the sex distorter transgene with the Y-chromosome^14^ (Fig. 3c) would ensure that the distorter increases in frequency with each generation since it would be present in all male progeny, resulting in a self-perpetuating genetic trait that could rapidly invade and suppress, perhaps eliminate, a natural population even if initially seeded at a very low frequency^15^,^16^,^17^,^18^.

## Methods

### gRNA design

Part of the 28S rDNA consensus sequences (~1 kb) of *Anopheles gambiae sensu stricto*, *Anopheles arabiensis*, *Anopheles merus, Anopheles melas* and *Anopheles bwambae* were aligned to identify regions of complete conservation across the *Anopheles gambiae species* complex but absent from the rDNA of distantly related *Anopheles stephensi*, *Anopheles funestus*, *Aedes Aeypti*, *Aedes Albopictus* and *Drosophila melanogaster*. ZiFiT (http://zifit.partners.org/) and ChopChop (https://chopchop.rc.fas.harvard.edu) CRISPR gRNA analysis tools were used to select three different gRNAs to specifically target rDNA sequences conserved across the gambiae complex (rDNA-T1 GGGTACAAGCTTGCGTACGTCGG, rDNA-T2 GCTTGTCCGACCGTGAGCCGTGG and rDNA-T3 GGATCCGTAACTTCGGGACAAGG) (Supplementary Fig. S1) and to test for absence of off-targets within *An. gambiae sensu stricto* genome. gRNA-T1 GGGTACAAGCTTGCGTACGTGTTTTAGAGCTAGAAATAGCAAGTTAAAATAAGGCTAGTCCGTTATCAACTTGAAAAAGTGGCACCGAGTCGGTGC targeting the rDNA-T1 locus was used for all experiments.

### Generation of the CRISPR^SD^ construct

**Figure 1:**
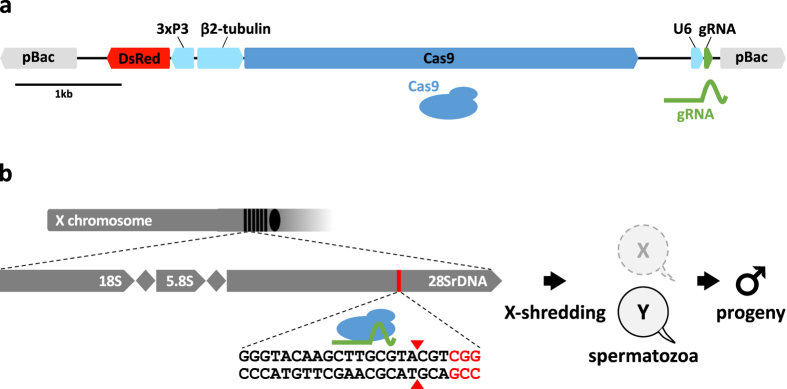
Generation of a CRISPR-Cas9 sex-ratio distortion system. (**a**) CRISPR^SD^ transformation construct. (pBac) *piggyBac* inverted repeats; (3xP3::DsRed) Pax promoter driving the DsRed marker to select gene integration events; (β2::Cas9) *Streptococcus pyogenes* Cas9 nuclease under the control of the male germline specific β2 tubulin promoter; (U6::gRNA) gRNA under the control of the ubiquitous U6 Pol III promoter. **(b)** Schematic representation of rDNA clusters within the *Anopheles gambiae* X-chromosome and the location of the multicopy gRNA target site. By shredding the X-chromosome during meiosis it is predominantly Y-bearing sperm that fertilize eggs generating a male-biased progeny.

The human codon-optimized version of the *Streptococcus pyogenes* Cas9 coding sequence was amplified from pX330 (AddGene, Zhang laboratory) using primers containing SalI (aacgtcgacGATCCCGGTGCCACCATGGA) and PacI (aacttaattaaTTTCGTGGCCGCCGGCCTTTT) restriction sites and subcloned into a vector containing the β2 tubulin promoter and terminator. AgeI and AscI were used to clone the β2::Cas9 expression cassette into the p165 vector containing the U6::spacer cloning site^8^, all flanked by *piggyBac* inverted repeats to obtain the p167 vector. BsaI Golden Gate cloning was used to insert the rDNA-T1 gRNA sequence GGGTACAAGCTTGCGTACGT after oligo-annealing of the primers T1F-TGCTGGGTACAAGCTTGCGTACGT and T1R-AAACACGTACGCAAGCTTGTACCC to obtain the final CRISPR^SD^ transformation construct (Fig. 1a).

### Generation and characterization of transgenic mosquito lines

Embryos from the strain G3 of *An. gambiae sensu stricto* (referred as wild-type) were injected with a mixture of 0.2 μg μl^−1^ of the CRISPR^SD^ construct and 0.4 μg μl^−1^ of a vasa-driven *piggyBac* transposase helper plasmid^19^ using a Femtojet Express injector and a Narishige 202ND micromanipulator mounted on a Nikon TE-DH100W inverted microscope. The hatched larvae were screened for the transient expression of the DsRed marker on a Nikon inverted microscope (Eclipse TE200) at a wavelength of 563 nm (Filter 630/30 nm emission, 595 nm dichroic). DsRed positive mosquitoes were individually crossed to wild-type to obtain transgenic lines. Strains originated from a single integration event were selected by the inheritance pattern of the DsRed marker scored in the progeny and confirmed by inverse PCR (Supplementary Table S2) as previously described^3^.

### Crosses and phenotype assays

**Figure 2:**
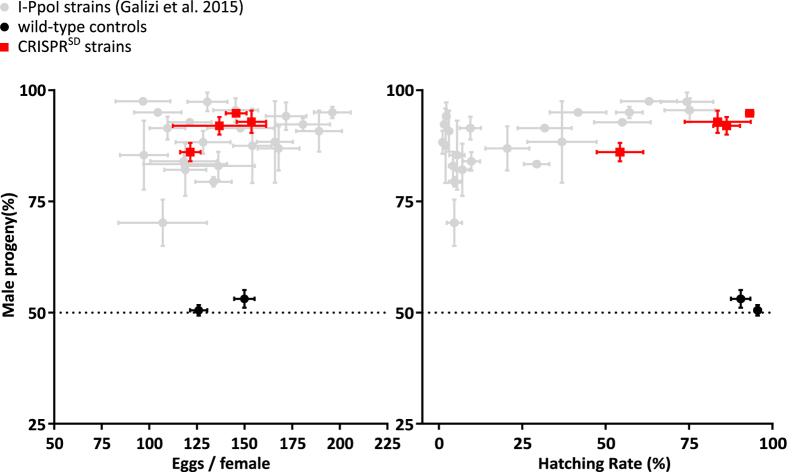
Crosses between CRISPR^SD^ males and wild-type females showing high sex-ratio distortion and fertility. The adult sex ratio of the progeny of hemizygous CRISPR^SD^ males (red squares) versus hemizygous I-PpoI males (grey dots, *Galizi et al*.^3^) and wild-type males crossed to wild-type females (black dots) with the average number of eggs per female (left panel) and the average hatching rate (right panel) shown. Error bars represent the standard error of the mean (SEM).

Fertility and adult sex ratio were assessed by crossing male mosquitoes heterozygous for the *CRISPR*^*SD*^ allele to an equal number of wild-type mosquitoes for 5 days. Wild-type males were crossed to equal number of wild-type females as a control. On the sixth day female mosquitoes were blood fed on an anaesthetised mouse and placed individually into 300 ml beakers 3d later. Females were given up to three days to oviposit into a 25 ml cup filled with water and lined with filter paper. For each deposition, the number of eggs laid as well as the larvae hatching were counted. The larvae were reared to adulthood and the total number of male and female mosquitoes were subsequently counted (Fig. 2, Supplementary Table S2). To determine whether the sex distortion phenotype was stably inherited from CRISPR^SD^ fathers, transgenic sons were crossed to wild-type females for up to five consecutive generations (Supplementary Table S2). In order to assess the paternal carry-over of the CRISPR-Cas9 components into the fertilized embryos, 10 CRISPR^SD^ males from strain I were crossed to the same number of vasa::Cas9 females and vice versa (vasa::Cas9 strain obtained from Eric Marois Lab). After individual ovipositions, number of eggs and hatched larvae were counted. Segregation of 3xP3::DsRed and 3XP3::YFP (Filter 535/30 nm emission, 515 nm dichroic) markers were used to screen respective segregation of the *CRISPR*^*SD*^ and *vasa::Cas9* alleles in first instar larval progeny (Supplementary Fig. S4).

### RT-PCR analysis of Cas9 expression in CRISPR^SD^ strain N and I

The terminal segment containing the gonads was dissected from the rest of the body from 10 transgenic adults for each sample. The remaining carcasses were used as a control sample. Total RNA was extracted using TRI reagent (Ambion) and reverse-transcribed using Superscript II (Invitrogen) after TURBO DNA-free (Ambion) treatment following the manufacturer’s instructions. Multiplex RT–PCR assay was performed on cDNA using fast-cycling PCR master mix (Qiagen) following the manufacturer’s instructions. In the same reaction mix we added primers specific for the Cas9 gene hCas9qF3-AAAGACCGAGGTGCAGACAG and hCas9qR3-CGATCCGTGTCTCGTACAGG as well as primers specific for the ubiquitous ribosomal gene RpS7 S7fwd2-GGCGATCATCATCTACGTG and S7rev2-GTAGCTGCTGCAAACTTCG (Supplementary Fig. S3).

### rDNA analysis

Genomic DNA was isolated from individual female mosquitoes originated from crosses between CRISPR^SD^ males and wild-type females using the Wizard Genomic DNA Purification Kit (Promega). Genomic loci containing the CRISPR^SD^ target sites were amplified with Phusion HF polymerase (Thermo Scientific) using RG236-GCAGCATAGGTGGGAGGGCTTCCTC and RG238-GTTTCTGTCCACACTGAGCTGACCT primers. Four-fifth of each PCR product were purified (Qiagen PCR purification kit) and digested with 1 μl of FastDigest Pfl23II (Thermo Fisher Scientific) for 1 hour at 37 °C. After heat inactivation at 65 °C for 15 min, the digested products were analysed on a 2% agarose gel as well as the remainder of each undigested PCR product (Supplementary Fig. S5).

## Additional Information

**How to cite this article**: Galizi, R. *et al*. A CRISPR-Cas9 sex-ratio distortion system for genetic control. *Sci. Rep*. **6**, 31139; doi: 10.1038/srep31139 (2016).

## Supplementary Material

Supplementary Information

## Figures and Tables

**Figure 3:**
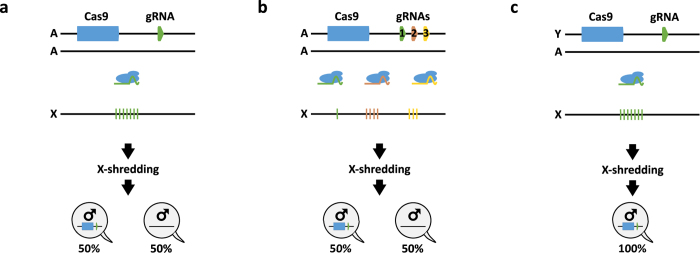
CRISPR^SD^ system: design and applications. (**a**) The RNA-guided Cas9 is expressed from an autosomal location and gives male bias by shredding a species-specific repetitive gene sequence conserved on the X-chromosome (any endonuclease such as TALENs, ZFN or HEG could be suitable for the purpose). The X-shredding construct is inherited only by half of the progeny and it would require inundative releases to suppress or eliminate a pest population^13^. (**b**) CRISPR-Cas9 can be multiplexed to target conserved gene sequences present in one or few copies on the X-chromosome^10^,^11^,^12^. (**c**) The endonuclease is placed onto the Y-chromosome and is therefore inherited by the entire male offspring. In doing so, even a small scale release would be self-sustaining and supress the population^15^,^16^,^17^,^18^.
