# Supplementary material for: A CRISPR-Cas9 sex-ratio distortion system for genetic control
Source: Sci Rep. 2016 Aug 3;6:31139. doi: 10.1038/srep31139 (PMC4971495; doi:10.1038/srep31139)
Supplement: Supplementary Information [file srep31139-s1.pdf]

A CRISPR-Cas9 sex-ratio distortion system for genetic control

Roberto Galizi[1] & Andrew Hammond[1], Kyros Kyrou[1], Chrysanthi Taxiarchi[1,2], Federica Bernardini[1], Samantha M. O'Loughlin[3], Philippos-Aris Papathanos[2], Tony Nolan[1], Nikolai Windbichler[1], Andrea Crisanti[1].

[1]Department of Life Sciences, Imperial College London, South Kensington Campus, London SW7 2AZ, UK. [2]Section of Genomics and Genetics, Department of Experimental Medicine, University of Perugia, 06132 Perugia, Italy. [3]Department of Life Sciences, Imperial College London, Silwood Park, Ascot SL5 7 PY, UK.

Supplementary Figure S1: CRISPR-gRNAs design.

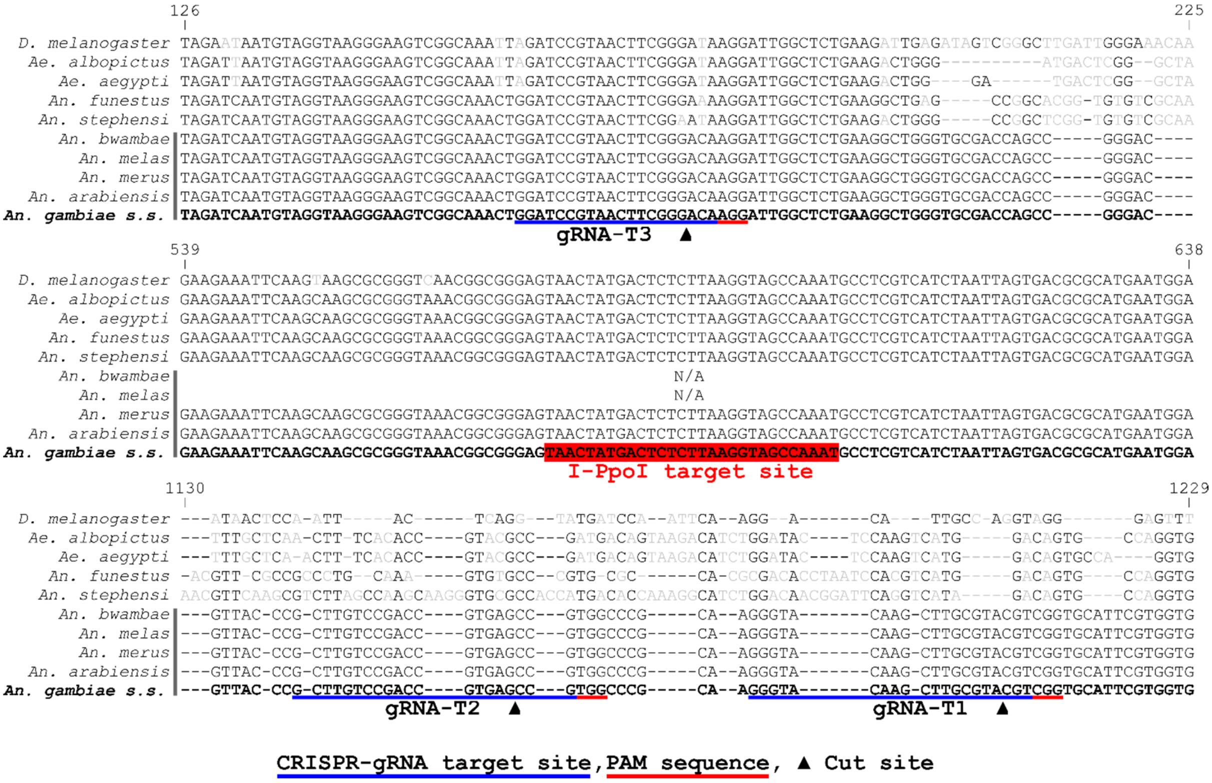

Nucleotide alignment of the 28S rDNA genes belonging to the Anopheles species *An. gambiae sensu stricto*, *An. arabiensis*, *An. merus*, *An. melas*, *An. bwambae, An. stephensi* and *An. funestus* (*the* grey bar indicates members of the *gambiae* species complex) as well as *Ae. Aeypti*, *Ae. Albopictus* and *D. melanogaster* (the three relevant sections of the 1 kb sequences are shown). CRISPR gRNA targets are underlined in blue, underlined in red is the protospacer adjacent motif (PAM) and the CRISPR-Cas9 cut site is indicated by a black arrowhead. Highlighted in red is the I-PpoI target site. gRNA-T1, was selected for its lack of off-targets sites within the *An. gambiae sensu stricto* genome. N/A indicates data not available.

Supplementary Table S2: Outcome of crosses between CRISPR[SD] transgenic and wild-type mosquitoes.

Transgenic line	Parental generation	CRISPR[SD] crossed to wild-type	# Eggs / female[1]	% eggs hatching[2]	% transgenic[3]	% male sex ratio[4]	Genomic position of insertion
I	G_1	♂	ND	ND	47.8 (113)	92.0 (112)	3L: 18751990
	G_2	♂	ND	ND	55.6 (138)	92.9 (311)	
	G_3	♂	ND	ND	47.8 (201)	88.1 (604)	
	G_4	♂	135.0 ±11.3 (675)	92.9	50.7 (513)	94.5 (513)	
	G_5	♂	147.7 ±6.1 (3693)	93.0	ND	94.7 (2070)	
N	G_1	♀	ND	ND	52.3 (111)	50.5 (111)	2L: 34761202
	G_2	♂	ND	ND	44.7 (78)	87.9 (141)	
	G_3	♂	ND	ND	44.6 (213)	89.3 (709)	
	G_4	♂	121.3 ±8.5 (485)	38.8	62.3 (143)	84.9 (146)	
	G_5	♂	121.5 ±6.2 (2673)	55.9	ND	87.2 (1175)	
B	G_1	♂	ND	ND	68.6 (35)	100 (32)	2L: 38962003
	G_2	♂	ND	ND	52.3 (287)	95.5 (134)	
	G_3	♂	ND	ND	56.2 (249)	96.4 (668)	
	G_4	♂	136.8 ±24.5 (547)	86.3	49.9 (339)	92.3 (339)	
G	G_1	♀	ND	ND	51.2 (129)	54.0 (124)	2L: 11862280
	G_2	♂	ND	ND	50.0 (772)	90.8 (772)	
	G_3	♂	ND	ND	51.5 (204)	89.8 (880)	
	G_4	♂	153.6 ±7.7 (768)	86.8	48.4 (517)	92.8 (517)	

Control crosses		# Eggs / female[1]	% eggs hatching[2]	% male sex ratio[4]
**wild-type ♂** x **wild-type ♀**	1	125.9 ±4.5 (3902)	96.2	50.5 (2840)
	2	149.9 ±5.4 (5096)	90.1	53.7 (583)

Analysis of the progeny of hemizygous CRISPR^{SD} individuals (females are indicated by a grey background) crossed to wild-type for 4 independent insertion events (I, N, B and G). As a control, wild-type females were crossed to wild-type males for two independent generations. (1) Average number of eggs recovered from single individual egg-deposition (± standard error of the mean; SEM). (2) Percentage of larvae hatching from the total number of eggs. (3) Percentage of transgenic offspring. (4) Percentage of males scored in the progeny. The total number of eggs (1) or individuals (3 and 4) counted in each cross is given in parentheses. Data not determined are indicated as ND.

Supplementary Figure S3: Cas9 mRNA expression in CRISPR^{SD} strain N and I.

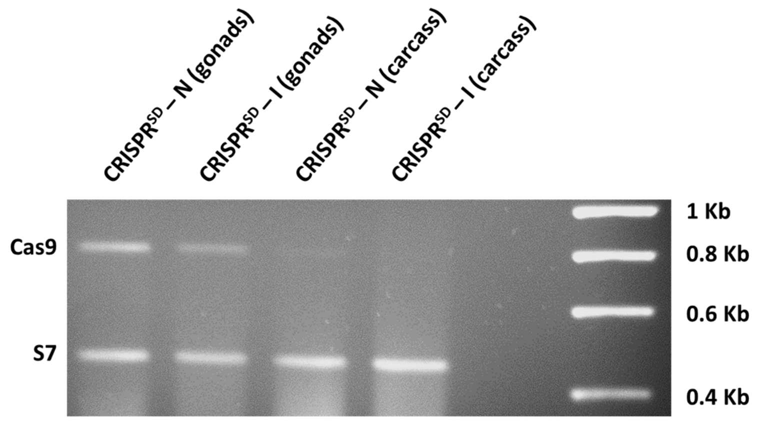

RT-PCR to detect Cas9 mRNA expression was performed on testes and carcasses dissected from CRISPR^{SD} transgenic males (strain N and I). Amplification of the ubiquitous ribosomal gene RpS7 was used as a control. A slightly more abundant Cas9 expression in testes together with residual unspecific expression detected in the carcass sample may explain the fertility reduction affecting strain N.

Supplementary Figure S4: Outcome of crosses between CRISPR^{SD} and vasa::Cas9 mosquitoes.

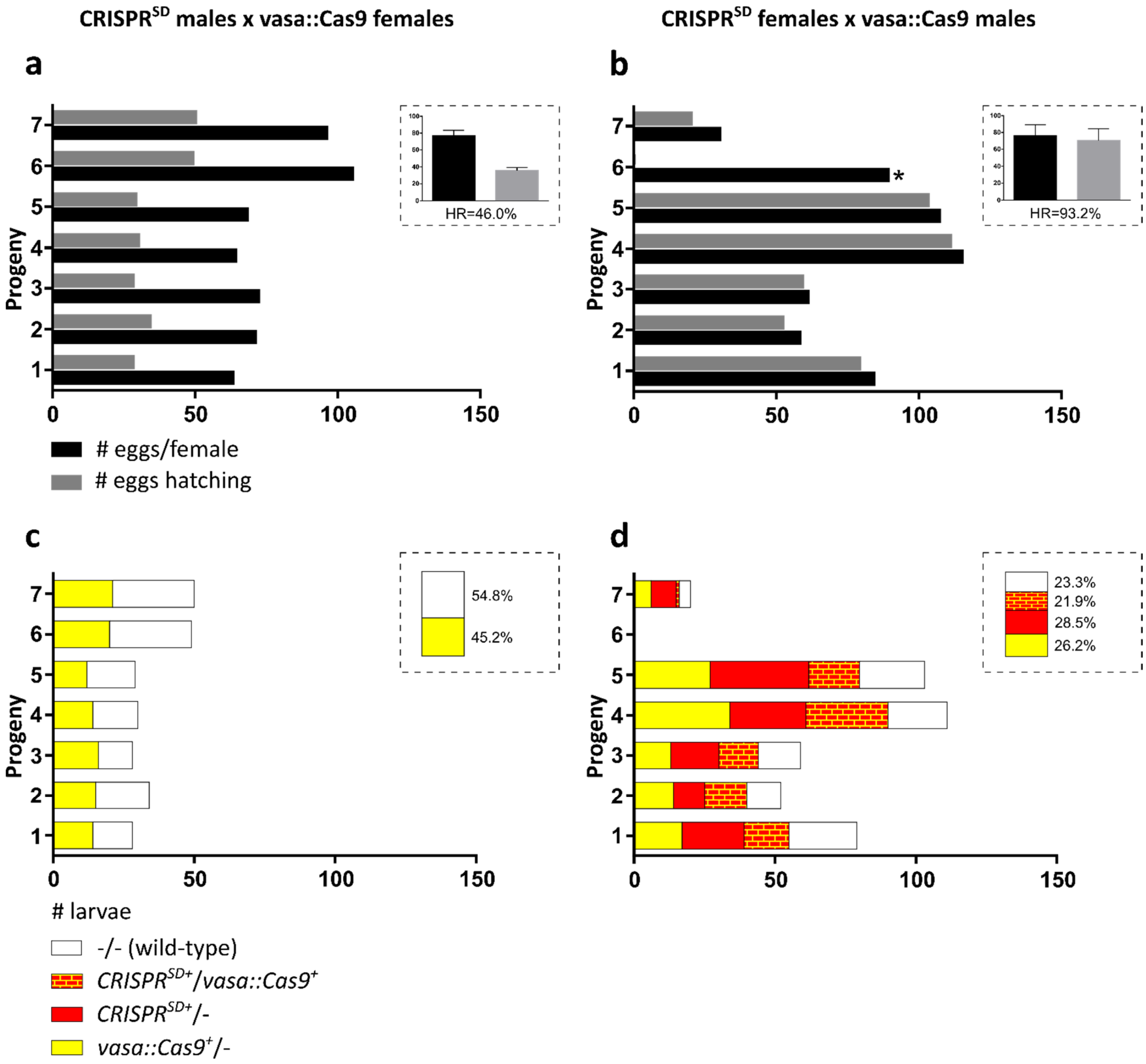

In order to assess carry-over of the Cas9 protein or the gRNA component by male sperm into the fertilized embryo, the CRISPR^{SD} strain I was crossed to a vasa::Cas9 strain that express *Streptococcus pyogenes* Cas9 under the *vasa2* promoter active in the germline of both sexes[1]. **(a-b)** Reciprocal crosses of the CRISPR^{SD} strain to the vasa::Cas9 strain. The number of eggs and larvae were obtained from 7 individual ovipositions (* indicates that the sample was removed from the average HR because of unknown mating status of the female). Average number of eggs per female, eggs hatching and overall hatching rate percentage (HR) are shown within the dashed insets. Error bars represents the SEM value. **(c-d)** Number of wild-type and transgenic individuals recovered from first instar larvae in the progenies. Total percentage of wild-type and transgenic larvae are shown within the dashed insets.

A reduction by half of the number of larvae hatching from CRISPR^{SD} males crossed to vasa::Cas9 females (a) compared to the reverse cross (b) accompanied by the total absence of *CRISPR^{SD}* alleles within the first instar larval progeny (c) is consistent with embryonic expression of the U6 driven gRNA and predominant maternal deposition of active *vasa2* driven Cas9 protein. No paternal carryover followed by *CRISPR^{SD}* allele killing was observed when vasa::Cas9 expressing males were crossed to CRISPR^{SD} females (b and d).

Supplementary Figure S5: Re-cleavage assay at the rDNA-T1 gRNA target site.

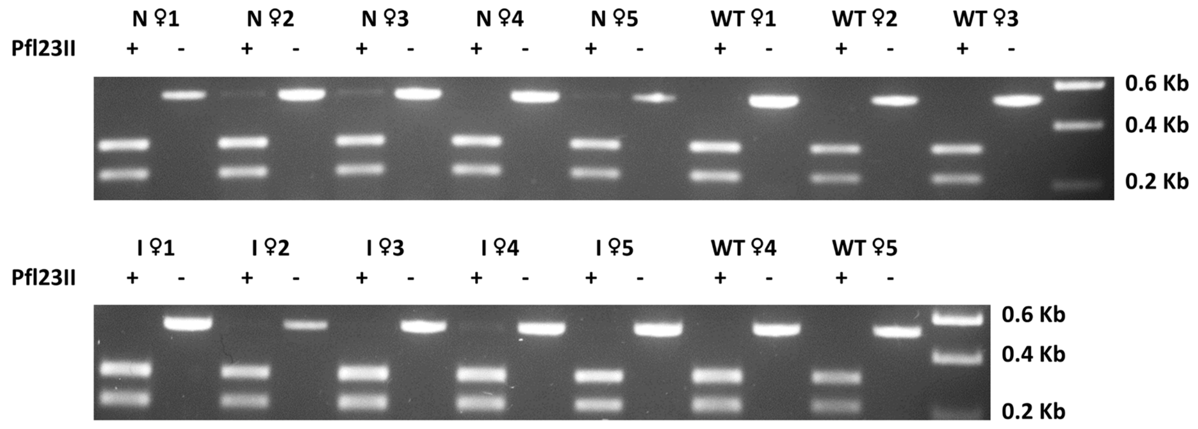

A total of 5 daughters of CRISPR^{SD}-N fathers (N ♀) and 5 daughters of CRISPR^{SD}-I fathers (I ♀) were individually analysed for modification to the gRNA target site on the ribosomal DNA repeats. 5 wild-type females were used as comparison. The genomic region encompassing the rDNA-T1 gRNA target site was amplified by PCR and the relative product digested *in-vitro* by using the Pfl23II restriction enzyme targeting the sequence CGTA^CG (^ indicates the position of the rDNA-T1 gRNA cut). In some of the CRISPR^{SD} individuals (N ♀2, N ♀3, N ♀5, I ♀2, I ♀4 and I ♀5) a small fraction of the PCR product remained resistant to the cleavage as a possible consequence of DNA misrepairs whilst a predominant portion of the product remained susceptible to cleavage.

1 Papathanos, P. A., Windbichler, N., Menichelli, M., Burt, A. & Crisanti, A. The vasa regulatory region mediates germline expression and maternal transmission of proteins in the malaria mosquito Anopheles gambiae: a versatile tool for genetic control strategies. *BMC molecular biology* **10**, 65, doi:10.1186/1471-2199-10-65 (2009).
